# Risk Factors Associated with Vancomycin-Resistant *Enterococcus* in Intensive Care Unit Settings in Saudi Arabia

**DOI:** 10.1155/2013/369674

**Published:** 2013-08-20

**Authors:** Mahmoud Shorman, Jaffar A. Al-Tawfiq

**Affiliations:** ^1^Internal Medicine Department/Infection Control Section, King Fahad Specialist Hospital, P.O. Box 15215, Dammam 31444, Saudi Arabia; ^2^Specialty Internal Medicine Unit, Dhahran Health Center, Dhahran 31311, Saudi Arabia

## Abstract

*Background*. Vancomycin-resistant enterococci (VRE) are significant nosocomial pathogens worldwide. There is one report about the epidemiology of VRE in Saudi Arabia. *Objective*. To determine the risk factors associated with VRE infection or colonization in intensive care unit (ICU) settings. *Design*. This is a descriptive, epidemiologic hospital-based case-control study of patients with VRE from February 2006 to March 2010 in ICU in a tertiary hospital in Saudi Arabia. *Methods*. Data were collected from hospital records of patients with VRE. The main outcome measure was the adjusted odds ratio estimates of potential risk factors for VRE. *Results*. Factors associated with VRE included ICU admission for multiorgan failure, chronic renal failure, prior use of antimicrobial agents in the past three months and before ICU admission, gastrointestinal oral contrast procedure, and hemodialysis. Being located in a high risk room (roommate of patients colonized or infected with VRE) was found to be protective. *Conclusions*. Factors associated with VRE acquisition are often complex and may be confounded by local variables.

## 1. Introduction

 Vancomycin-resistant enterococcus (VRE) is an important pathogen among hospitalized patients. Significant morbidity, mortality, and increased hospital costs have been associated with infections due to VRE [1]. Detection of new cases of VRE represents cross transmission via the hands of health care workers, contaminated equipment, and environmental surfaces [2]. The emergence of de novo VRE through genetic mutations induced by glycopeptide exposure in an individual patient is unusual [3]. Acquiring nosocomial VRE may vary according to how endemic VRE is in a specific location, the exposure to contaminated equipment, VRE carrier proximity referred to as “colonization pressure,” and patient's hospitalization duration which is referred to as the “time at risk.” Colonization pressure is defined as the proportion of patients colonized with a particular organism in a defined geographic area within a hospital during a specified time period [4]. Differentiating among the factors associated with nosocomial spread of VRE or amplification of previously undetectable colonization is difficult in clinical settings [5].

 The first report of VRE from Saudi Arabia was in 1993 from King Faisal Specialist Hospital-Riyadh [6]. However, there are only three studies of VRE from Saudi Arabia. One study described the frequency of VRE as normal flora of the intestine in Saudi patients. Of 4276 patients, VRE (*E. faecium*) was found in six patients [7]. The second study characterized 34 vancomycin-resistant VanA *E. faecium* isolates obtained from two hospitals in Saudi Arabia [8]. The third study describes the prevalence and risk factors for fecal carriage in patients at tertiary care hospitals. In that study, only 7 out of 157 rectal swabs obtained from patients in different clinical setting were VRE positive [9].

Here, we report the result of the surveillance study of VRE in a Saudi Arabian hospital and describe the associated risk factors for VRE colonization and infection in our region. The goal of this study was to identify significant risk factors for acquiring VRE colonization and infection in ICU settings using a case-control study.

## 2. Methods

This is a retrospective, case-control study of VRE cases at King Fahad Specialist Hospital-Dammam, a referral hospital providing tertiary care for the province of Dammam, Saudi Arabia. The hospital has 18 medical-surgical intensive care unit beds, and more than 6000 patients are admitted to King Fahad Specialist Hospital-Dammam annually.

## 3. Population

Records obtained from the Infection Control Section and the Clinical Microbiology Laboratory were reviewed to identify ICU patients who had VRE (*E. faecalis or E. faecium*) isolated from either surveillance cultures or clinical specimens between February 2006 and March 2010. King Fahad Specialist Hospital-Dammam does have guidelines for wide screening of new hospital admission for MRSA and VRE to prevent outbreaks of these infections. Intensive care unit, for example, performs active surveillance for methicillin-resistant *Staphylococcus aureus* (MRSA) and VRE at ICU admission. 

### 3.1. Data Collection

Medical records of patients with and without VRE were reviewed, and the following information was collected (as outlined in Table 2).

1. Demographic data (age, gender).
2. Host-related factors (ICU admission, acute renal failure, sepsis or multiorgan failure, and underlying diseases).
3. Hospital related factors: referral from other hospitals; hospital admission in the previous year; length of stay of previous year's hospitalization ICU length of stay.
4. Medication-related factors: use of antimicrobial agents in the past three months, duration of antibiotic use, use id corticosteroid, chemotherapeutics, and cyclosporine.

### 3.2. Definitions

VRE colonization or infection date is defined as the date on which a positive sample was collected. Recent antimicrobial use was defined as receipt of any antimicrobial agent for more than 3 consecutive days in the 3 months before the date of culture detection; patients who received short courses of perioperative prophylaxis were excluded by this criterion. Renal insufficiency was defined as a creatinine concentration greater than 1.7 mg/dL. High risk ICU room is a room of previous patients colonized or infected with VRE. 

### 3.3. Microbiology 

#### 3.3.1. Culture Method, Conventional Organism Identification and Susceptibility Testing

Rectal swabs for culture were first inoculated onto *Columbia PNBA* and then into salt broth. Plates were incubated at 35°C in ambient air and examined for growth at 24 and 48 hours. Any suspected colonies were identified by conventional laboratory methods, including Gram stain, catalase test, BEA test, and BVS (vancomycin screening agar that incorporates the use of 6 ug/mL of vancomycin in brain-heart infusion agar) [10]. Black colonies (esculin positive) were then subcultured onto a blood agar plate for purity. Following 24 hours incubation, a definite spot of growth or greater than one colony present at the site of inoculation on the BVS agar indicates that the Enterococci may be a VRE [11]. Identification (*E. faecalis/E. faecium*) was confirmed by performing Gram-positive (GP) identification card on the vitek 2 system (bioMerieux; GP colorimetric identification card). Susceptibility testing was performed on confirmed enterococcal isolates using vancomycin (0.016 to 256 *μ*g/mL) and teicoplanin (0.016 to 256 *μ*g/mL) *E* test strips. The determination of the MICs and the interpretation of vancomycin resistance (MIC ≥ 32 *μ*g/mL) were done according to the Clinical and Laboratory Standards Institute (CLSI) guidelines [12]. For the interpretation of the teicoplanin results, combination of the intermediate and resistant MICs was done as previously published for the assignment of isolates as having VanA (MIC ≥ 16 *μ*g/mL) or VanB (MIC < 16 *μ*g/mL) [13].

#### 3.3.2. Case Control

Patients who had VRE colonization or infection (30 cases) were matched 1 : 2 to randomly selected controls who were patients in the same ward or unit during the study period. Controls were selected in such a way that the distributions of case patients and control patients were similar over the dates of hospitalization. The controls were selected from the population of patients whose surveillance or clinical culture findings were negative for VRE. During the study period, there were 2200 surveillance cultures obtained, and only 30 (1.4%) distinct cultures were positive. Data collection for controls was performed as it was for case patients. 

### 3.4. Data Analysis

We encoded all data into a database and used Stata (version 7) for analysis. We compared the characteristics of cases and controls using the chi-square test for categorical variables and the *t*-test for continuous variables. To investigate which potential risk factors were associated with VRE, we performed unconditional logistic regression with adjustment for age, sex, and ward. Odds ratios (ORs) and corresponding 95% confidence intervals (CIs) were used as summary statistics to assess risk. A *P* value of .05 or less was considered statistically significant. Multivariate analysis was conducted to determine the potential risk factors for acquisition of VRE.

## 4. Results

Between February 2006 and March 2010, a total of 30 patients in ICU were identified with VRE colonization or infection. They were randomly matched to 60 controls.  Table 1 shows the characteristics of cases and controls. As a result of the matching process, the distributions of cases and controls were similar in terms of age, sex. 

The case patients were more likely to have multiorgan failure upon ICU admission (33% versus 12%, *P* = 0.03), more likely having underlying chronic renal failure (43% versus 15%, *P* < 0.01), receiving hemodialysis (37% versus 18%, *P* = 0.05), or receiving GI contrast procedure (17% versus 2%, *P* = 0.03). Case patients were more likely to have received antimicrobial agents in the 3 months before the study period (69% versus 20%, *P* < 0.01) especially vancomycin, metronidazole, quinolones, and piperacillin-tazobactam. Being on chemotherapeutic agents was observed in 10.7% of VRE-positive versus 1.6% of VRE-negative patients (*P* value = 0.09). Of interest, it was found that being located in a high risk room (roommate of patients colonized or infected with VRE) was protective (Table 2). Multivariate analysis showed that prior antibiotic use was an independent determinant for the acquisition of VRE (*P* = 0.026).

## 5. Discussion 

Vancomycin-resistant enterococcus is becoming the causative agent in an increasing number of health-care-associated infections in the last decade especially in the United States [14]. Nowadays, VRE is reaching Middle East countries like Saudi Arabia, and to our knowledge, this is the second published study on the epidemiology and risk factors of VRE from Saudi Arabia. Also, Vancomycin-resistant enterococci are becoming more important for hospital-infection control, mostly due to their particular features: colonization of the gastrointestinal tract, difficulty in decolonization of patients, and the environment dissemination [15].

Case-control study was performed comparing all known risk factors for VRE colonization from the current literature, including high risk host with comorbidities including underlying diseases like chronic renal failure, diabetes mellitus, ischemic heart disease, congestive heart failure, liver failure, and liver cirrhosis; hospital-related factors including hospital admission in previous year including ICU, length of hospitalization, having special procedures during hospitalization, and insertion of devices; and medication- related factors like the use of antimicrobial agents in the past three months [5, 15, 16]. Univariate analysis of our data suggested that the potential risk factors for the new detection of VRE were multiorgan failure upon ICU admission, more likely having underlying chronic renal failure or receiving hemodialysis, was more likely to have received antimicrobial agents in the three months before the study period especially vancomycin, metronidazole, quinolones, and piperacillin-tazobactam. These data are consistent with those of previous reports [17, 18]. The association of colonization with renal failure suggests that patients who are more ill are more vulnerable to colonization with VRE.

Numerous studies of both colonized and infected patients explored the role of preceding antimicrobial treatment as a risk factor for nosocomial VRE with conflicting results. It was suggested that previous use of vancomycin, cephalosporins, and antimicrobial agents with an antianaerobic spectrum is important in the development of VRE [5].

 Our study showed similar data regarding prior exposure to vancomycin, metronidazole, piperacillin-tazobactam, and quinolones as a major risk factor for the development of VRE. It is interesting to note that being located in a high risk ICU room (roommate of patients colonized or infected with VRE) was protective. This finding may be explained by the strict isolation precautions taken in these settings. However, it was shown previously that roommates of patients identified as colonized or infected with VRE were at substantial risk of becoming colonized, with the degree of risk increasing in older and more frail patients [19]. Molecular typing of the isolates from this outbreak revealed that the predominant VRE comprised 20 VanB, five VanA, and one VanA/VanB type isolates, which tended to fall into two genetic clusters that were identifiable phenotypically by their susceptibility to tetracycline [20].

In conclusion, the factors associated with acquisition of VRE are often complex, may be confounded by local variables, and may be different depending on whether the patient acquires VRE by nosocomial transmission or by primary in vivo emergence (e.g., gene transfer to previously susceptible enterococci). In addition, VRE seems to be uncommon in Saudi Arabia. Our study suggests that strict infection control and isolation procedures are effective in controlling health-care-associated transmission of VRE, as it was shown that being in a high risk room (room of previous patients colonized or infected with VRE) was protective. This observation is likely related to more vigilant postdischarge cleaning and disinfection of these rooms. One limitation of the study is the small sample size, but VRE is not common in Saudi Arabia.

## Figures and Tables

**Table 1:** Characteristics of VRE cases and controls.

Variable	Cases (*n* = 30)	Controls (*n* = 60)	*P*
Male sex	18 (60%)	35 (58%)	0.88
Age (mean, SD)	62.8, 21.0	62.0, 19.7	0.85

**Table 2:** The risk factors that were found significantly associated with VRE on univariate analysis.

Risk factor	Cases (*n* = 30)	Controls (*n* = 60)	Adjusted odds ratio* (93% CI)		*P* value
Host-related factors					
ICU admission due to multiorgan failure	33%	12%	5.4%	(1.2–4.9)	<0.01
Underlying chronic renal failure	43%	15%	4.6%	(1.6–3.0)	<0.01
Medication-related factors					
Use of antimicrobial agents in past 3 months	69%	20%	11.7%	(3.6–38.1)	<0.01
Use of pre-ICU antibiotics	77%	37%	5.6%	(2.1–15.9)	<0.01
Vancomycin	32%	4%	12.7%	(1.3–119.8)	0.03
Metronidazole	59%	24%	5.0%	(1.4–17.3)	0.01
Piperacillin-tazobactam	87%	27%	17%	(2.9–98.4)	<0.01
Quinolones	54%	18%	5.8%	(1.5–22.1)	0.01
Hospital-related factors					
High risk room	71%	98%	0.04%	(0.004–0.4)	<0.01
GI contrast procedure	17%	2%	12.5%	(1.3–117.6)	0.03
Hemodialysis	37%	18%	2.9%	(1.0–8.5)	0.05

*Adjusted for age, sex.
